# Connecting the nucleus to the tip: A protein module linking RSL4 transcription with ROP signaling in root hair growth

**DOI:** 10.1093/plcell/koag097

**Published:** 2026-03-27

**Authors:** Luciano M Di Fino

**Affiliations:** Assistant Features Editor, The Plant Cell, American Society of Plant Biologists; Umeå Plant Science Centre (UPSC), Department of Forest Genetics and Plant Physiology, Swedish University of Agricultural Sciences, Umeå 901 83, Sweden

Plant roots enhance water and nutrient uptake by forming root hairs, which are specialized extensions of epidermal cells on the root surface (Gilroy and Jones 2000). In Arabidopsis, root hair development begins when an epidermal cell receives positional signals from the underlying cortex. These signals specify a precise site on the cell surface and trigger the outgrowth of an initial bulge. Root hair formation relies on a coordinated signaling network that drives tip growth, which is a rapid and highly polarized mode of cell expansion. ROP-family small GTPases play a central role by establishing a tip-focused signaling domain that organizes the actin cytoskeleton and directs secretory trafficking to the apical plasma membrane (Wu et al. 2000). At the same time, a sharp apical Ca^2+^ gradient and locally produced reactive oxygen species work alongside actin dynamics to maintain vesicle trafficking, remodel the cell wall, and sustain directional elongation at the growing tip (Preuss et al. 2004).

It is important to note that not all epidermal cells form a root hair, because this process requires the activation of specific genes. Over the last decades, researchers have identified many root hair–specific genes expressed in epidermal cells. The core bHLH transcription factor ROOT HAIR DEFECTIVE6 (RHD6) and its homologs, the RHD6-LIKE (RSL) genes, function as master regulators of root hair development (Yi et al. 2010). RHD6 activates early in a subset of epidermal cells and promotes root hair development largely by inducing RSL4 and RSL2 (Yi et al. 2010). In turn, RSL4 drives a broad root hair growth program by upregulating cell wall–modifying and tip growth–associated genes that support elongation. Despite extensive characterization of RSL4-mediated transcriptional regulation and ROP signaling in root hair growth, the mechanisms that integrate these pathways to coordinate root hair development remain unclear.

In recent work, Meng Xu and colleagues (Xu et al. 2026) studied the genetic regulation of root hair development. Through transcriptomic analyses, they found that 2 members of the BOUNDARY OF ROP DOMAIN (BDR) family, BDR6 and BDR7, are enriched in root hairs. Motivated by this observation, the authors set out to define the roles of BDR6 and BDR7 during root hair development.

Using reverse genetics, the authors showed that *bdr6* and *bdr7* single mutants showed reduced root hair density and shorter root hairs. The *bdr6 bdr7* double mutant showed an even stronger reduction in both density and length, indicating an additive effect (Fig. 1a). These results support that BDR6 and BDR7 contribute to normal root hair initiation and elongation. Spatiotemporal expression analysis by confocal microscopy in epidermal cells, using the reporter lines BDR6g:GFP and BDR7g:GFP, showed that BDR6/7 localize to the apical plasma membrane of root hairs, consistent with their proposed role as ROP effectors. The reporters also showed nuclear localization, suggesting an additional role in transcriptional regulation (Fig. 1b). Interestingly, apical plasma membrane association depends on ROP signaling, as it is lost in the *rop2;rop4i;rop6* mutant, whereas nuclear localization remains unaffected.

**Figure 1:**
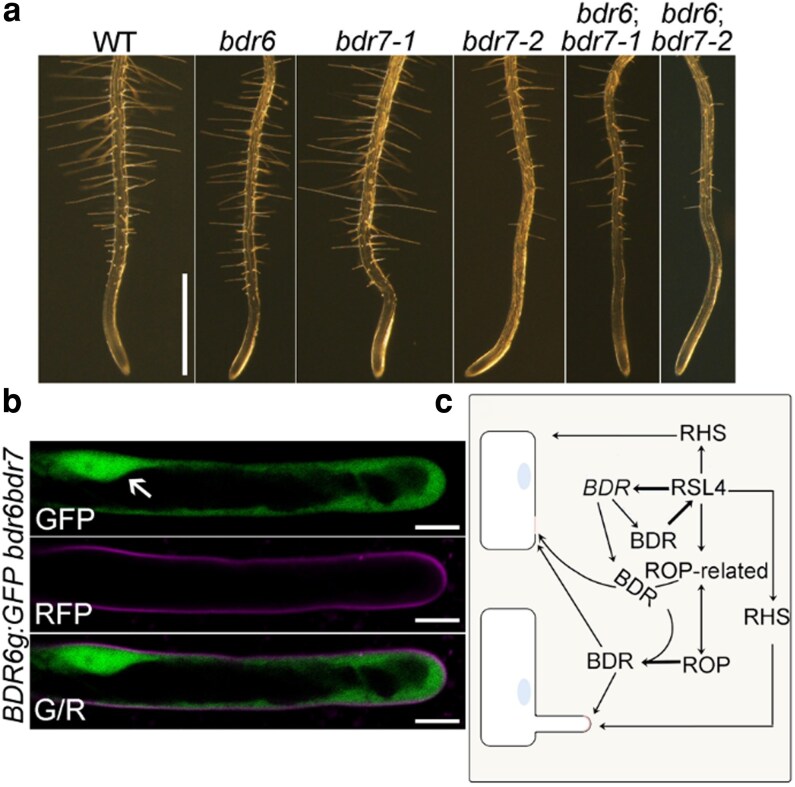
BDR6/7 promote root hair development through interaction with RSL2/4. a) Root hair phenotype in WT and *bdr6*, *bdr7-1*, *bdr7-2*, *bdr6bdr7-1*, and *bdr6bdr7-2* mutants. b) Confocal image showing the subcellular localization of BDR6 (green). Magenta shows the cell wall, stained with propidium iodide. The white arrow indicates nuclear localization. c) Diagram summary role of BDR, RHS, RSL4, and ROP in root hair initiation and elongation. Figure adapt from Xu et al. 2026, Figs. 2 and 10.

To better understand the nuclear role of BDR6/7, the authors tested whether BDR6/7 physically interact with RSL2/4, given their roles as master regulators during root hair initiation. Using yeast 2-hybrid assays, they detected positive interactions between BDR6/7 and RSL2/4. This finding raised the question of whether they act in the same regulatory pathway. Overexpressing BDR6 or BDR7 in wild type increased both root hair density and length, but this elongation-promoting effect was lost in the *rsl2rsl4* double mutant.

To support the idea that BDR6/7 work with the RSL pathway to control gene expression, the authors used a dual-luciferase assay: coexpressing RSL4 with BDR6 or BDR7 increased reporter activity compared with RSL4 alone, indicating that BDR6/7 enhance RSL4-driven transcription. Together, these results suggest that BDR6/7 help RSL4 efficiently activate genes required for root hair development.

Finally, they found that RSL4 binds to specific regulatory regions of BDR6/7 in ChIP-qPCR assays. These results point to a positive-feedback loop in which RSL4 induces BDR6/7, and BDR6/7 in turn enhance RSL4-driven transcription.

Using reverse genetics, microscopy, and molecular assays, Xu and colleagues propose that BDR proteins play an unexpected role in transcriptional regulation during root hair development. Their data support a positive feedback loop in which BDR6/7 interact with RSL2/4 and reinforce the RSL-driven gene expression program. Despite these interesting findings, there are lots of compelling questions. How is the dual distribution of BDR6/7 regulated? What cis- and trans-regulatory factors mediate their dual distribution? What intracellular activities are controlled by the ROP-BDR6/7 module at the apex? These questions await future investigation.

## Recent related articles in *The Plant Cell*

- Li and Zhu (2024) showed that the RHO-RELATED PROTEIN FROM PLANTS 2 (ROP2) mRNA is required for polarized root hair growth and analyzed how the untranslated regions (UTRs) are necessary for correct mRNA localization and translation at the apical tip of the root hair.
- Ke et al. (2025) found that the wheat awn inhibitor gene B1 limits root hair length by repressing reactive oxygen species signaling.

## Data Availability

No new data were generated or analysed in support of this research.
